# Microbial Grazers May Aid in Controlling Infections Caused by the Aquatic Zoosporic Fungus *Batrachochytrium dendrobatidis*

**DOI:** 10.3389/fmicb.2020.592286

**Published:** 2021-01-21

**Authors:** Hazel N. Farthing, Jiamei Jiang, Alexandra J. Henwood, Andy Fenton, Trent W. J. Garner, David R. Daversa, Matthew C. Fisher, David J. S. Montagnes

**Affiliations:** ^1^Shanghai Universities Key Laboratory of Marine Animal Taxonomy and Evolution, Key Laboratory of Exploration and Utilization of Aquatic Genetic Resources, National Demonstration Center for Experimental Fisheries Science Education, Shanghai Ocean University, Shanghai, China; ^2^Department of Evolution, Ecology and Behaviour, Biosciences Building, University of Liverpool, Liverpool, United Kingdom; ^3^Institute of Zoology, Zoological Society of London, London, United Kingdom; ^4^MRC Centre for Global Infectious Disease Analysis, Imperial College London, London, United Kingdom

**Keywords:** ciliates, disease, fungi, microbial loop, protozoa, *Tetrahymena*

## Abstract

Free-living eukaryotic microbes may reduce animal diseases. We evaluated the dynamics by which micrograzers (primarily protozoa) apply top-down control on the chytrid *Batrachochytrium dendrobatidis* (*Bd*) a devastating, panzootic pathogen of amphibians. Although micrograzers consumed zoospores (∼3 μm), the dispersal stage of chytrids, not all species grew monoxenically on zoospores. However, the ubiquitous ciliate *Tetrahymena pyriformis*, which likely co-occurs with *Bd*, grew at near its maximum rate (*r* = 1.7 d^–1^). A functional response (ingestion vs. prey abundance) for *T. pyriformis*, measured using spore-surrogates (microspheres) revealed maximum ingestion (*I*_*max*_) of 1.63 × 10^3^ zoospores d^–1^, with a half saturation constant (*k*) of 5.75 × 10^3^ zoospores ml^–1^. Using these growth and grazing data we developed and assessed a population model that incorporated chytrid-host and micrograzer dynamics. Simulations using our data and realistic parameters obtained from the literature suggested that micrograzers could control *Bd* and potentially prevent chytridiomycosis (defined as 10^4^ sporangia host^–1^). However, simulated inferior micrograzers (0.7 × *I*_*max*_ and 1.5 × *k*) did not prevent chytridiomycosis, although they ultimately reduced pathogen abundance to below levels resulting in disease. These findings indicate how micrograzer responses can be applied when modeling disease dynamics for *Bd* and other zoosporic fungi.

## Introduction

Although the traditional microbial food web (i.e., prokaryotes and protists, *sensu* Azam et al. (1983) is well-established as a driver of aquatic productivity (Fenchel, 1987), fungi are only now being appreciated as integral aquatic microbes. A dominant group of fungi, the chytrids, are parasites of phytoplankton, zooplankton, and vertebrates (Kagami et al., 2014), and zoospores, the chytrid dispersal stage, are an appropriate size for protozoan grazers and are highly nutritious, containing essential fatty acids and sterols (Fenchel, 1987; Gleason et al., 2008; Kagami et al., 2014; Gerphagnon et al., 2018; Frenken et al., 2020a). Hence, through top-down control micrograzers within the microbial food web have the potential to reduce the likelihood or severity of, or even prevent, disease outbreaks caused by these pathogens (Kagami et al., 2014; Grossart and Rojas-Jimenez, 2016; Frenken et al., 2019). Here, by developing and parameterizing a population model we explore the dynamics by which microbial grazers may control the chytrid *Batrachochytrium dendrobatidis*, a panzootic pathogen of amphibians that is argued to have caused the greatest loss of biodiversity attributed to any disease (Scheele et al., 2019).

**FIGURE 1:**
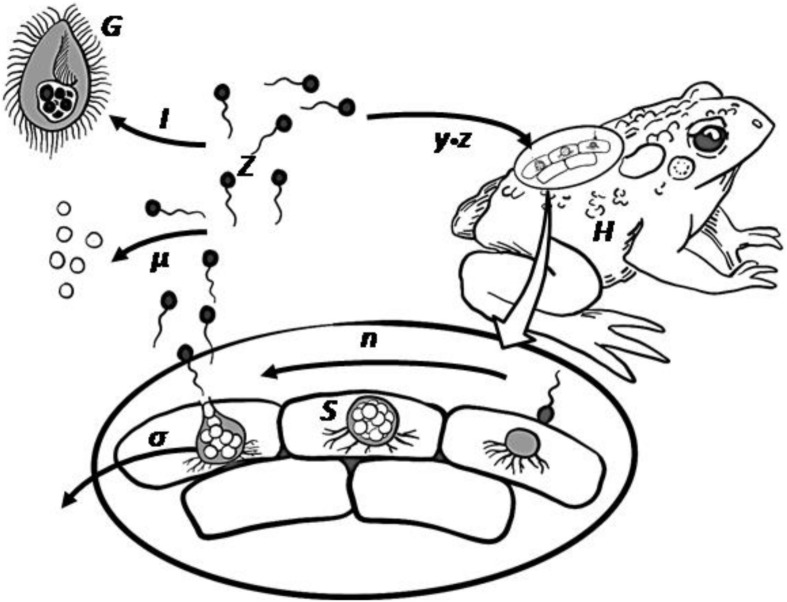
*Bd* infectious life cycle including the potential grazing pressure by micrograzers. Zoospores (*Z*) move using a flagellum, and on contact infect the amphibian host (*H*). Within the host epidermal cells, a spore then forms a sporangium (*S*) that releases further zoospores through asexual reproduction (*n*), after which the sporangium dies (*σ*). Released zoospores may die naturally (*μ*) or be ingested (*I*) by micrograzers (*G*).

*Batrachochytrium dendrobatidis* (henceforth, *Bd*) infects amphibian hosts through the dispersal of motile, 3–5 μm zoospores (Figure 1). The environmental pool of zoospores is instrumental in driving infection dynamics, as these can accrue in a dose-dependent manner (Garner et al., 2009), with for some hosts the size of the zoospore pool influencing long-term consequences for population survival or extinction (Briggs et al., 2010). It follows that processes that reduce the zoospore-pool should reduce the probability and intensity of disease outbreaks. Consumption of zoospores by naturally occurring micrograzers has been suggested to result in losses sufficient to reduce infections. Experiments show that some micrograzers may reduce the likelihood of *Bd* infections, and field data indicate a negative relationship between potential-grazer abundance and both the prevalence of infection and host mortality from disease (Woodhams et al., 2008; Buck et al., 2011; Gleason et al., 2014; Schmeller et al., 2014). There is now a need to build on these observations and investigate in more depth the dynamics by which consumers may impact on *Bd*.

Studies investigating the spores-grazer link, the “mycoloop,” have shown that multiple micrograzers can survive and grow on chytrid zoospores, including rotifers and copepods (Kagami et al., 2007, 2014; Schmeller et al., 2014). However, most work to date on the consumption of *Bd* zoospores has focused on large zooplankton, especially cladocerans (Buck et al., 2011; Hamilton et al., 2012; Searle et al., 2013; Maier et al., 2016). However, experiments on cladocerans have used unrealistically high micrograzer abundances (>10–100 times higher than natural levels), and at natural levels large zooplankton seem to have little impact on *Bd* infections (Buck et al., 2016). Micrograzers, in contrast, are abundant in shallow waters and are often near the bottom of ponds where infected hosts (e.g., benthic, grazing tadpoles) spend time resting and grazing on the substrate (Dionne, 1969; Beiswenger, 1977; Thurnheer and Reyer, 2001). Furthermore, as many protozoa have generation times on the order of hours, by reproducing asexually when zoospores are abundant, micrograzer populations may increase several fold, consuming zoospores as they are released from the host. We, therefore, argue that protozoa will be more important than cladocerans in reducing the abundance of chytrid zoospores. This is supported by Schmeller et al. (2014) who, using mesocosms, showed the ciliate *Paramecium* can significantly reduce the number of hosts infected with *Bd* by up to 65% when it is introduced at naturally occurring abundances.

We also suggest that the main impact of micrograzers on *Bd* spore-load will be in the water directly surrounding the host, where zoospores will be most abundant. Field studies suggest that in water bodies where *Bd* occurs, zoospore densities in the water column are low, ranging from ∼0.5 to 500 L^–1^ (Kirshtein et al., 2007; Walker et al., 2007). In contrast, zoospores are shed from hosts at up to 250 zoospores min^–1^ (Maguire et al., 2016), surviving only ∼1–3 days (Woodhams et al., 2008). Additionally, zoospores mostly disperse on the order of 1 cm (Piotrowski et al., 2004), demonstrating chemotaxis toward keratinised skin over this distance (Moss et al., 2008; Garmyn et al., 2012). Although these laboratory-based rates will be dependent on environmental factors such as temperature (Woodhams et al., 2008), they suggest that the limited movement and survival of the rapidly produced zoospores will lead to dense aggregations in localized regions around the host. Recognizing the likelihood of these local abundances and the well-established density-dependent feeding and growth responses of micrograzers (Fenchel, 1987), in this study we focused attention on the impact of micrograzers on *Bd* dynamics around the host. We achieved this by: first, investigating a range of potential micrograzers, determining which survived on a diet of only *Bd* zoospores and if they grew at their maximum rates when fed saturating prey concentrations – we then concentrated on those species that grew; second, measuring ingestion and growth rates of a common, widely distributed species that thrives on *Bd*; and third, using these data, developing and exploring a model that couples the *Bd* life-cycle with micrograzer-control on zoospores. In doing so, we indicate the dynamics by which micrograzers may reduce *Bd* populations – potentially preventing disease – and provide a mechanism by which chytrid-diseases can be incorporated into microbial food web models.

## Materials and Methods

### Culture Maintenance

*Batrachochytrium dendrobatidis* (*Bd*) cultures (strain #262 IA 9′13, Imperial College London) were maintained at 18°C (at which all experiments were conducted) on H-broth medium (500 mL: 5 g Tryptone and 16 g glucose) and were regularly transferred (every ∼5 days) to maintain exponential growth. Bacterial growth was prevented by the addition of antibiotics (Ampicillin at 100 μg ml^–1^; Kanamycin at 50 μg ml^–1^; Chloramphenicol at 34 μg ml^–1^). Note, antibiotics may have small detrimental effects on protozoan swimming speed (Wu et al., 1996), but we assume here that under saturating food conditions they will have a negligible impact on growth. Micrograzers were obtained from Sciento (Manchester, United Kingdom): the ciliates *Blepharisma* sp., *Oxytricha* sp., *Paramecium aurelia*, *Paramecium caudatum*, *Stentor coeruleus, Tetrahymena pyriformis*, and *Urocentrum turbo* and the rotifers *Brachionus calyciflorus* and *Philodina* sp. *Tetrahymena pyriformis* was maintained axenically for extended periods on H-broth. All other species were maintained prior to experiments on a natural assemblage of bacteria in Chalkley’s medium enriched with cereal grains, as provided by Sciento (Chalkley, 1930).

### Assessing Growth of Micrograzer Species on *Bd* Zoospores

**FIGURE 2:**
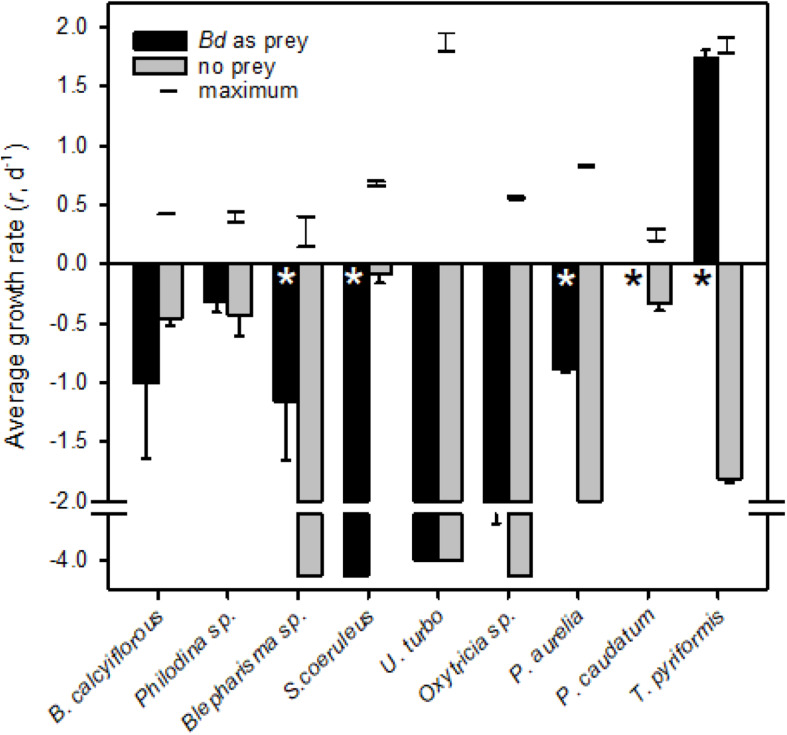
Average growth rates (*r*, d^− 1^) for micrograzers fed *Bd* (black) or no prey (gray); error bars are one standard error, and * indicates where significant (α = 0.05) differences occurred between fed and unfed treatments. All grazers exhibited a constant growth or death rate except from the two Paramecium species which we expand on in Figure 3. Note that for *P. caudatum* growth rate was zero when fed *Bd*. The horizontal lines connected by a vertical line represent the range of predicted maximum growth rate (i.e., food saturated on suitable prey) at 18°C, for each species. Maximum growth rate data were obtained from various sources at a range of temperatures (Kasturi Bai et al., 1969; Finlay, 1977; Taylor, 1978; Jackson and Berger, 1984; Inamori et al., 1990; Weithoff and Walz, 1995; Weisse et al., 2013; Salt et al., 2017) and were converted to rates at 18°C by two methods, either assuming a Q_10_ of 2 or that growth rate varies linearly with temperature at a rate of 0.07 *r* (d^− 1^) °C^− 1^ (Montagnes et al., 2003).

We tested the hypothesis that *Bd* zoospores were of nutritional benefit to the micrograzer. To do so, we compared potential maximum growth (i.e., at expected saturating prey concentrations, that may be reached near the host Weisse, 2017) on zoospores to when no food was available (i.e., when grazers would be starving). We also compared growth on zoospores to maximal rate of growth of the micrograzers, obtained from literature estimates (Figure 2).

Prior to introducing micrograzers, *Bd* was isolated from its growth medium to ensure that the medium was not a source of nutrients for the micrograzers. To do so, a *Bd* suspension (in exponential phase) was centrifuged (50 ml tubes, 5000 rpm, 6 min), the supernatant removed, and the pellet resuspended in autoclaved Volvic^®^ mineral water to a concentration >1.50 × 10^5^ ml^–1^ (determined microscopically). Resuspended cultures were examined microscopically (40×) to ensure zoospores were motile. Bacterial growth was prevented with antibiotics, as above.

To assess growth rate, we followed our previous methods (Montagnes, 1996). Micrograzers (9 species) were passed 5 times through autoclaved Volvic^®^ water to remove bacteria. Then 5 to 8 individuals, dependent on micrograzer size, were added to a 10 ml well containing the *Bd* suspension. Parallel treatments containing only sterile Volvic^®^ water were used to assess mortality rate in the absence of prey, i.e., starvation rates. All treatments (i.e., species incubations with or without *Bd*) were replicated in triplicate (i.e., three 10 ml wells). To assess growth rate (*r*, d^–1^), the number of gazers in each well was determined microscopically, using a dissection microscope, after 2 or 3 days (depending on the observed change in abundance). Then, new *Bd* suspensions were prepared (as above), and micrograzers were transferred to these, maintaining *Bd* abundance. If micrograzer numbers decreased (net death occurred) over the incubation, then all individuals were transferred, but if numbers increased (net growth occurred) then the initial number was transferred. Cells death was estimated by their disappearance (Montagnes, 1996). This procedure was continued for 14 days or until all micrograzers had died. Cultures were routinely checked under a compound microscope (100×), to ensure there was no bacterial contamination.

When numbers increased between transfers, growth rate (*r*, d^–1^) was determined over each incubation period, as *r* = *ln*(*N*_*t*_/*N*_0_)/*t*, where *N*_0_ and *N*_*t*_ are the micrograzer abundance on the initial and final day, respectively, and *t* is the incubation period (2 or 3 days); to determine growth rate across all transfers (up to 14 days), the average of these was obtained. When micrograzer numbers decreased between transfers, mortality rates (–*r*, d^–1^) were determined as slope of *ln* numbers over the entire incubation period. To assess if growth (or death) rate differed between treatments (i.e., with or without *Bd*) a two tailed *t*-test was conducted (α = 0.05).

### Quantifying the Functional Response (Ingestion vs. Prey Abundance) of *Tetrahymena* Grazing on *Bd*

Our study focused on *Tetrahymena pyriformis* as: (1) it grew rapidly on *Bd* zoospores (see section “Results”) and therefore clearly consumed and assimilated zoospores; (2) it is a model organism for which there are substantial data (see section “Discussion”); and (3) it is common, globally, in habitats where *Bd* may occur (see section “Discussion”). Prior to determining ingestion rate, *T. pyriformis* was acclimated with zoospores for >10 h. To do so, the ciliates were first removed from H-broth by centrifugation (50 ml tubes, 8000 rpm, 8 min) and then resuspended in 10 ml of autoclaved Volvic^®^ water. To obtain only zoospores, a centrifuged *Bd* culture (as above) was filtered through a 5 μm Nitex^®^ mesh. Zoospores were then added to the water containing ciliates, to a total volume of 20 ml (resulting in ∼10^6^ zoospores ml^–1^), with antibiotics (as above). This acclimation had no negative effects on the ciliates: after 10 h, zoospore abundance had substantially decreased and ciliate abundance increased (indicating the ciliates were feeding and growing), the ciliates behaved similarly to when grown on H-broth (i.e., similar swimming pattern), and their cell size and shape were similar to when grown on H-broth.

To determine ingestion rate on spore-sized particles, 3 μm fluorescent polymer microspheres (henceforth beads, Fluoro-Max^TM^, Thermo Fisher Scientific, United States) acted as a surrogate for *Bd* zoospores which are 3–5 μm (Berger et al., 2005). Bead concentrations, ∼8 × 10^3^ ml^–1^ to 10^6^ ml^–1^ (see section “Results”), were prepared in autoclaved Volvic^®^ water and vortexed prior to use, ensuring mono-dispersion. An aliquot (0.5 ml) of the acclimated ciliate culture (>30 micrograzers) was added to 1 ml of Volvic^®^ water with beads, at various concentrations [with more measurements at low abundances (Montagnes and Berges, 2004), see section “Results”], and incubated for 5 or 10 min, depending on the bead concentration (preliminary experiments deemed these to be appropriate incubation periods). Incubations were terminated by fixing cells with ethanol (final concertation 70%). The average number of beads ingested per ciliate (>30 cells) was determined via fluorescent microscopy and was subsequently used to determine ingestion rate (*I*, prey d^–1^) at each prey concentration.

The relationship between ingestion rate and zoospore abundance (*Z* ml^–1^), was determined by fitting a Type II functional response to the data: *I* = *I*_*max*_^∗^*Z*/(*k* + *Z*), where *I*_*max*_ (*Z* min^–1^) is the maximum ingestion rate and *k* is the half saturation constant (*Z* ml^–1^). The response was fit using the Marquardt-Levenberg algorithm (SigmaPlot, Systat, Germany); this algorithm is appropriate for describing such biological data sets (Berges et al., 1994).

### Modeling Micrograzer Impacts on *Bd* Populations

To assess the dynamics by which grazing pressure impacts on *Bd* infection populations we developed and applied the following model that couples a reduced version of the *Bd*-load model (Briggs et al., 2010) with the Rosenzweig-MacArthur predator-prey model (Turchin, 2003). Data for *T. pyriformis* were used to represent micrograzers (see the Discussion for a justification to focus on this species). Following logic outlined in the Introduction, the model describes the infection load on a single host and, as a proxy for the waters surrounding the host, only considers a volume of 10 ml around that host, where zoospores and micrograzers reside. As a metric to predict chytridiomycosis, it assumes that a sporangia load of 10^4^ per host results in host mortality (Briggs et al., 2010), with the recognition that this will vary between hosts and *Bd* strains (Fisher et al., 2009; Kilpatrick et al., 2010). It does not include reduction of spore numbers by emigration as zoospores are unlikely to disperse far before dying (Piotrowski et al., 2004), and we assume through chemokinesis, micrograzers remain near their food source (Leick and Hellung-Larsen, 1992; Montagnes et al., 2008; Walker et al., 2010). The model is described by the following equations,

(1) $$\frac{d\,S}{d\,t}=y\,v\,Z-\sigma \,S$$

(2) $$\frac{d\,Z}{d\,t}=\eta \,\text{S-yZ}-\mu \,Z-\frac{I_{m\,a\,x}\,Z}{k+Z}\,G$$

(3) $$\frac{d\,G}{d\,t}=e\,\frac{I_{m\,a\,x}\,Z}{k+Z}\,\text{G–dG}\,\left(1-\frac{b}{G}\right)$$

where for Eqs. 1, 2, *S* is the number of sporangia ml^–1^ (note for per host measurements this value is multiplied by 10); *Z* is the zoospore abundance (ml^–1^); *y* is the per capita spore-host encounter rate; *v* is the fractional likelihood of spore infection per encounter; *σ* is the per capita sporangia mortality rate; *η* is the per sporangia spore-release rate; and *μ* is the per capita spore mortality rate (see Table 1).

**TABLE 1:** Parameters used in the *Bd*-grazer model (Eq. 1–3), to assess temporal dynamics of *Bd* and grazer abundances (Figures 5, 6).

Symbol	Parameter	Estimate	Range explored by Briggs et al.	Dimension
*y*	Rate of zoospores encounter with hosts	(0.05)	Large range of values	d^–1^
*v*	Fraction of successful *Bd* spore infections	0.1	0–1	dimensionless
η	Production rate of zoospores from sporangium	17.5	–	d^–1^
σ	Sporangia loss rate	0.2	0.1–0.3	d^–1^
*μ*	Spore death rate	0.1	0.02–1	t^–1^
*E*	Conversion efficiency	5 × 10^–4^		dimensionless
*I*_*max*_	Maximum ingestion rate	1630		S d^–1^
*K*	Half saturation constant	5.75 × 10^4^		S ml^–1^
*d*	Micrograzer death rate	0.01		t^–1^
*b*	Minimum number of micrograzers	1		ml^–1^

**Bd* parameter estimates are from Briggs et al. (2010) who present a wide range of possible values; we have chosen one set of these that provide an illustration of Bd-micrograzer dynamics. Micrograzer data are from our functional response (Figure 4). Conversion efficiency (*e*) was estimated as described in the text. The minimum number of micrograzers (*b*) and micrograzer death rate (*d*) were estimated from personal observations.*

Then, Eqs. 2, 3 were coupled to include spore loss by micrograzers (*G*), where grazing (*I*) is dictated by the functional response (see *Tetrahymena pyriformis* ingestion, above); *e* is the abundance-based conversion efficiency, determined assuming a biomass-based estimate of *e* is ∼0.1 (Montagnes et al., 2019) and biovolumes of *Bd* zoospores and *T. pyriformis*; and *d* is the micrograzer per capita death rate. We assume here that *Bd* zoospores are not the only potential food source for the micrograzers, and so incorporate a minimum micrograzer abundance (*b*) that implicitly assumes that in the absence of zoospores the micrograzer population is maintained by the presence of other potential food sources; hence we model potential increases in micrograzer abundance over and above this minimum, dependent on consumption of *Bd* zoospores. Estimates of *d* and *b* are based on our unpublished observations when *Tetrahymena* was exposed to poor food conditions. Table 1 summarizes the above parameters and the estimates used.

All model runs (100 d) were initiated with 10 sporangia host^–1^ (1 sporangium ml^–1^), 100 zoospores ml^–1^, and 1 micrograzer ml^–1^ (again assumed to be the minimal population size, maintained by other resources in the environment). For *Bd*, we applied parameter values that were within the range explored by Briggs et al. (2010) (Table 1).

We first performed simulations to assess the ability of *T. pyriformis* to control *Bd*. Then, we assessed the extent to which micrograzers that are inferior to *T. pyriformis* could control *Bd*, through exploration of micrograzer parameter space. Inferior micrograzers had reduced maximum ingestion rate (up to 0.5 × *I*_*max*_ of *T. pyriformis*) and increased half saturation constant (up to 2 × *k* of *T. pyriformis*); see Figure 4 for an indication of the range of these responses. To quantify the impact of micrograzers on *Bd*, we examined the maximum abundance (over the 100 days) and the abundances at 50 and 100 days of *S*, *Z*, and *G*.

## Results

### Assessing Growth of Micrograzer Species on *Bd*

**FIGURE 3:**
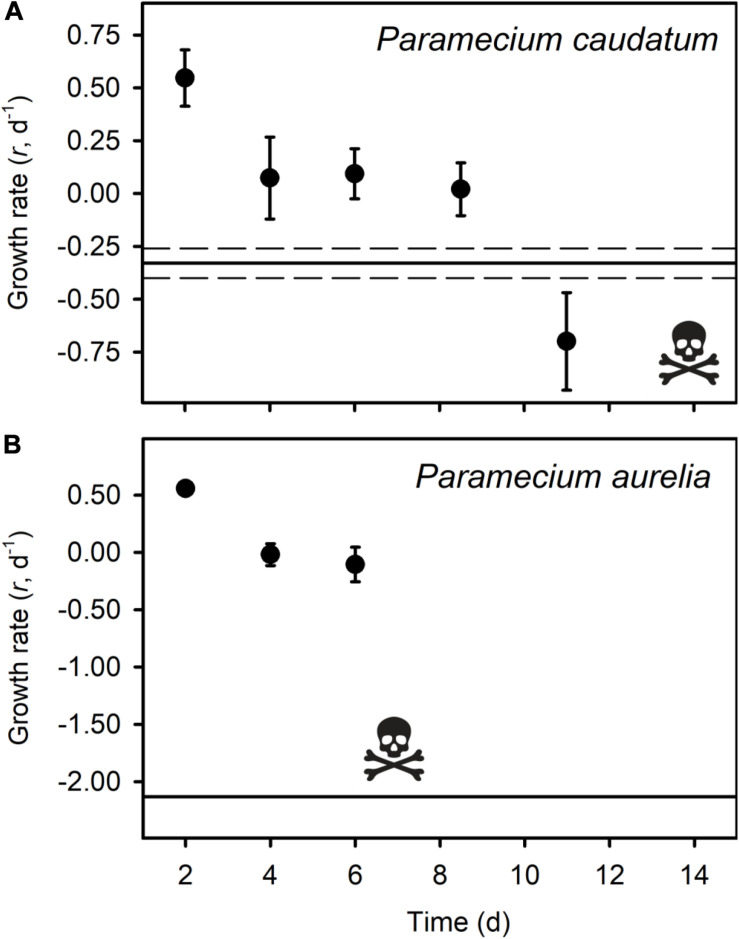
Average growth rates (*r* d^– 1^) of three replicates of *Paramecium caudatum* **(A)** and *Paramecium aurelia* **(B)** in the *Bd* treatment, with standard error bars. The skull and crossbones indicate the time point where all individuals had died. The solid black line represents the average death rate of the micrograzers when no prey were present, and the dotted black line indicates the standard error of the control groups.

**FIGURE 4:**
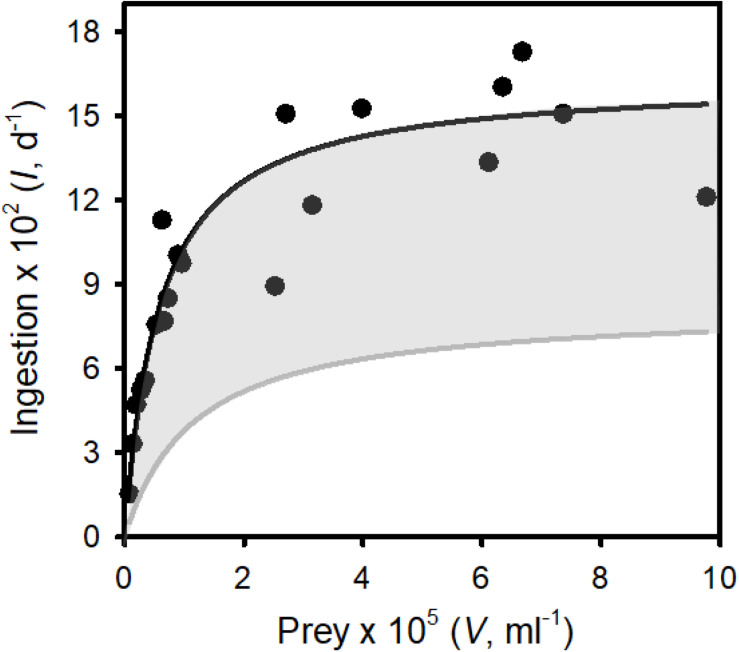
The functional response: ingestion rates of *Tetrahymena pyriformis* on surrogate zoospores (prey) vs. prey concentration. Points are ingestion rates at defined prey abundances. The solid line represents the best fit of a Type II functional response to the data (see Results for the parameter estimates). The gray region represents the range of functional responses used to assess the ability of “inferior micrograzers” to control *Bd* (i.e., reduced maximum ingestion rate and increased half saturation constant; see Methods, Modeling micrograzer impacts on *Bd* populations).

All of the micrograzers can be maintained in laboratory cultures, with maximum growth rates ranging from ∼0.4 to 2 d^–1^ (Figure 2), and all died when maintained on water alone, indicating their relative mortality rates when starved (Figure 2). When fed *Bd*, micrograzers exhibited four distinct responses (Figure 2): (1) for the ciliate *Stentor coeruleus* the death rate was significantly (and substantially) higher than in water alone; (2) for the ciliates *Urocentrum turbo, Blepharisma* sp., and *Oxytricha* sp. and the rotifer *Philodina* sp. there was no significant difference between death rate with or without *Bd*; (3) for the rotifer *Brachionus calyciflorus* growth rate initially increased (i.e., after 48 h) followed by death, and for the ciliates *Paramecium aurelia* and *P. caudatum* (Figure 3) the growth rate was initially positive when *Bd* was present followed by a negative growth rate as time progressed – on average over the incubation *P. aurelia* exhibited negative growth while *P. caudatum* exhibited zero growth (Figures 3, 4) for the ciliate *Tetrahymena pyriformis* there was a sustained positive growth rate (Figure 2), which was significantly higher than the negative growth rate on water alone; this growth rate of ∼1.7 ± 0.23 (SE) d^–1^ was equal to that when the ciliate was grown axenically on H-broth (unpublished data) and near its maximum rate under any conditions.

**FIGURE 5:**
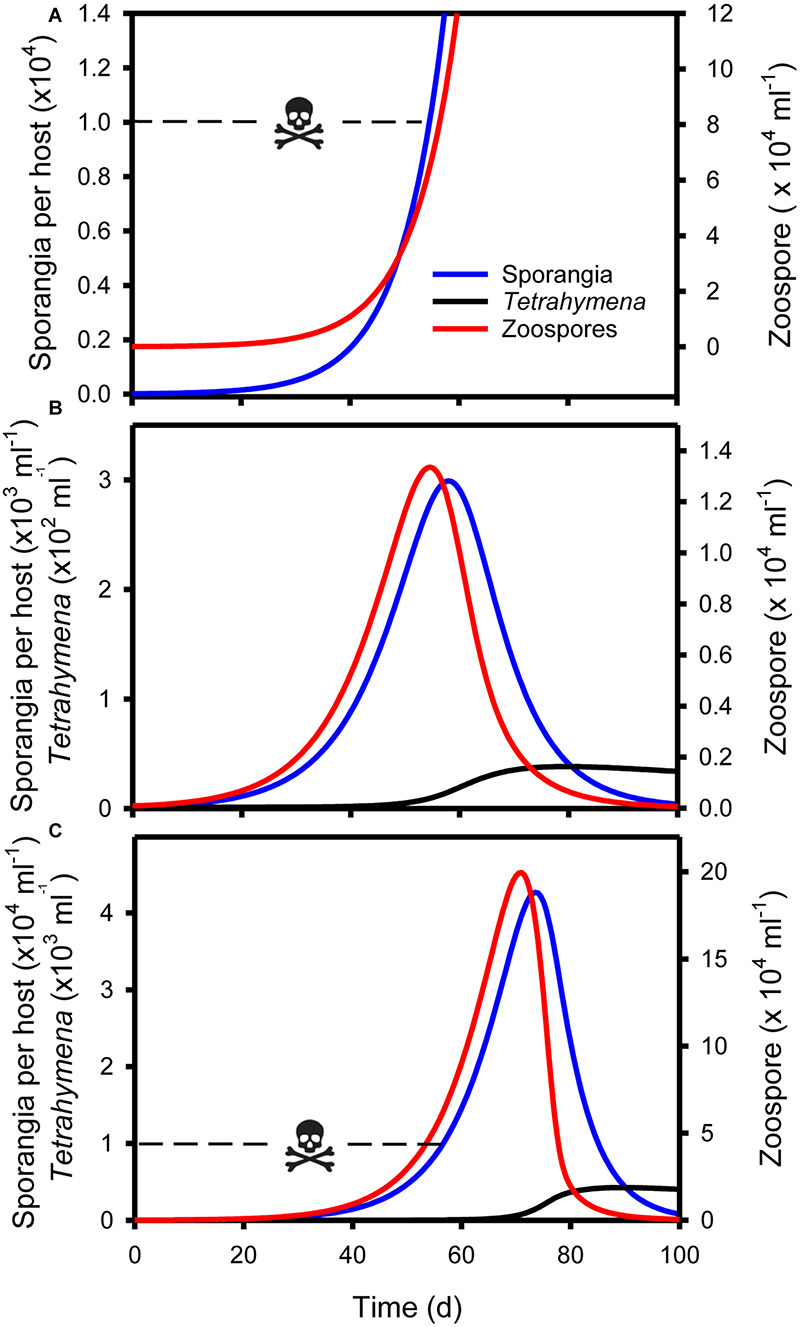
Simulations of micrograzer (*T. pyriformis*) control of *Bd*, based on Eq. 1–3 and parameters presented in Table 1. **(A)** *Bd* (zoospore and sporangia) dynamics in the absence of micrograzers, indicating that by ∼55 days sporangia per host approach lethal limits [skull and crossbones, 10^4^ sporangia per host Briggs et al. (2010)]. **(B)** *Bd* and micrograzer dynamics, indicating control of zoospores and sporangia, maintaining sporangia numbers below the lethal limit. **(C)** *Bd* and micrograzer dynamics based on an inferior micrograzer to *T. pyriformis* (0.5 × *I*_*max*_; 2 × *k* presented in Table 1), indicating the micrograzers inability to prevent host death at ∼55 days (skull and crossbones) but its ability to ultimately reduce *Bd* levels by 100 days.

**FIGURE 6:**
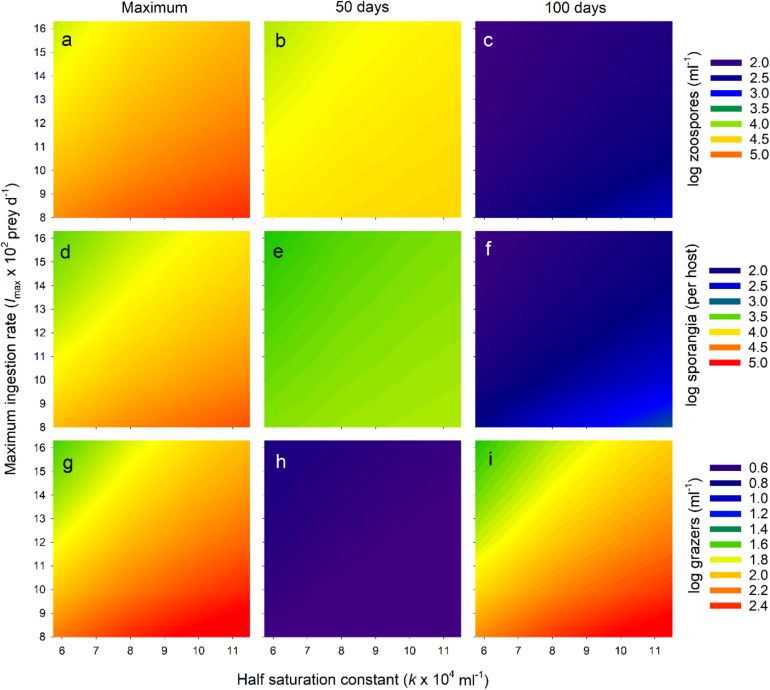
Exploration of *Bd*-micrograzer dynamics (Eq. 1–3), through varying two key micrograzer parameters: the half saturation constant (*k*) and the maximum ingestion rate (*I*_*max*_); see Methods for details. To characterize dynamics, we provide log numbers of zoospores **(a–c)**, sporangia **(d–f)**, and micrograzers **(g–i)**. Note that for each of these, abundance is presented as a colour-map, with the associated colour-key to the right of the associated three panels. To indicate trends, for each stage (zoospores, sporangia, grazers), we only present three abundances: the maximum number reached over the 100 days (Maximum), the number at 50 days (50 days), and the number at 100 days (100 days), presented in the first, middle and last columns, respectively.

### Quantifying the Functional Response of *Tetrahymena* Grazing on *Bd*

As *T. pyriformis* grew on zoospores alone it was clear that this ciliate ingested, digested, and assimilated *Bd* zoospores. Zoospores were also observed in *T. pyriformis*, in the food vacuoles of the ciliate, under 40 × magnification, further supporting their consumption. Ingestion rate using beads as a surrogate for *Bd* zoospores followed a typical Type II (rectangular hyperbolic) functional response (Figure 4, adjusted *R*^2^ = 0.82), with *I*_*max*_ = 1.63 × 10^3^ ± 98 (SE) prey d^–1^ and *k* = 5.75 × 10^3^ ± 1.38 × 10^3^ (SE) prey ml^–1^.

### Modeling Micrograzer Impacts on *Bd* Populations

Control of *Bd* occurred when parameters for the micrograzer (*T. pyriformis*) were included in the model (Figure 5). In the absence of the micrograzer, sporangia per host reached lethal levels [>10^4^ host^–1^ (Briggs et al., 2010)] by ∼55 days (Figure 5A). However, when micrograzers were included their population rose from 1 to ∼35 ml^–1^, with the result that sporangia were limited to a maximum of 3 × 10^3^ per host (i.e., based on the assumption that 10^4^ sporangia is a lethal limit, the host would survive), and *Bd* was virtually eradicated by 100 days (Figure 5B).

We then assessed the ability of micrograzers that were inferior to *T. pyriformis* to control *Bd*, through exploration of micrograzer parameter space: i.e., up to twice the half saturation (*k*) and half the maximum ingestion rate (*I*_*max*_) of *T. pyriformis* (Figure 4). Figure 5C illustrates population dynamics when the most inferior micrograzer was included (highest half-saturation constant and lowest maximum ingestion rate): the general pattern remained similar to that when *T. pyriformis* parameters were applied, with the micrograzers controlling *Bd* over 100 days, but the abundance of zoospores, sporangia, and micrograzers were more than 10 times greater than the simulation including *T. pyriformis*, leading to predicted host death at ∼55 days and a peak in abundance at ∼70 days.

We then examined the pattern of the temporal dynamics across a wider range of parameter space (representing a range of predators-types) by reporting the maximum abundance and the abundances at 50 and 100 days of zoospores, sporangia, and micrograzers. Across all parameters explored, the micrograzer population provided top-down control of *Bd*, as over the entire range *Bd* was virtually eradicated by 100 days (Figures 6C,F). However, the quantitative levels and rates of control varied considerably with micrograzer efficiency: with reduced *I*_*max*_ and increased *k*, zoospores and sporangia reached higher maximum abundances (Figures 6A,D) and persisted longer (Figures 6B,E), indicating a decrease in the control of *Bd*. In particular, micrograzers with <0.7 *I*_*max*_ (∼10^3^ prey d^–1^) and >1.5 × *k* (∼9 × 10^4^ ml^–1^) were not capable of preventing sporangia per host exceeding lethal levels of 10^4^ per host (the yellow-to-red region on Figure 6D). Decreased *I*_*max*_ and increased *k* also led to increases in micrograzer abundance (Figures 6G–I), in response to the increased spore levels available under these grazing regimes.

## Discussion

Control of a range of diseases caused by zoosporic fungi may be achieved through consumption of zoospores (Kagami et al., 2014; Grossart and Rojas-Jimenez, 2016; Frenken et al., 2019). Here, we explore the dynamics by which micrograzers may controlling the amphibian disease chytridiomycosis, caused by *Batrachochytrium dendrobatidis* (*Bd*). Several micrograzers are known to consume *Bd* zoospores (Schmeller et al., 2014); we expand on this by indicating that *Paramecium* spp. grow on *Bd* zoospores for short periods and the ubiquitous ciliate *Tetrahymena pyriformis* maintains near maximal rate on zoospores alone. These findings provide essential information for modeling population dynamics. We then determine the functional response of *T. pyriformis* feeding on spore*-*sized prey, which is also required for population modeling. Finally, using our data and literature estimates, we develop and employ a novel model that couples the *Bd* life cycle with micrograzer-control on zoospores. This synthesis reveals the dynamics by which micrograzers may suppress *Bd* loads and argues that to predict *Bd*-host interactions it will be useful to consider embedding these into the larger microbial food web.

### Micrograzer Growth on *Bd*

Micrograzers may consume *Bd* zoospores (Schmeller et al., 2014), and chytrid zoospores can be nutritious (Frenken et al., 2020a). All of the micrograzers we examined also could ingest *Bd*, but they exhibited a range of growth-responses (Figure 2). For one ciliate, *S. coeruleus*, *Bd* appeared to be toxic, possibly explaining reports that *S. coeruleus* does not reduce *Bd* viability (Schmeller et al., 2014), while other species seemed to obtain no nutritional benefit. However, several species benefited from ingesting *Bd*. Both *Paramecium aurelia* and *P. caudatum* initially grew, although this was not sustained (Figure 3), suggesting that while *Bd* is of some value, it may lack essential nutrients for these ciliates. In contrast, *Tetrahymena pyriformis* sustained positive growth, indicating that *Bd* can provide a complete diet for certain species. These observations are supported by previous work on ciliates: *T. pyriformis* and a closely related species, *Colpidium striatum*, also grow on yeast (*Saccharomyces*), while *P. aurelia*, and *P. caudatum* cannot, again possibly due to a lack of nutrients such as essential fatty acids and B-vitamins (Hirayama and Satuito, 1991; Pritchard et al., 2016).

Our analysis, therefore, suggest that not all micrograzers would be capable of or equally proficient at controlling *Bd*. However, with additional prey sources to sustain the consumers, there may be selective feeding on *Bd*. For instance, although some studies have shown *Tetrahymena* to be a non-selective feeder (Kirby et al., 2019) others show that *T. pyriformis* differentiates between bacterial prey, leading to a more efficient assimilation of prey biomass and a greater cell yield of ciliates (Thurman et al., 2010). Likely, in the mesocosm experiments conducted by Schmeller et al. (2014), where *Paramecium* controlled *Bd*, this ciliate’s diet was supplemented by naturally occurring bacteria. In fact, in our initial growth-experiments, where antibiotics were not included, bacteria grew, and *Paramecium caudatum* consumed zoospores in addition to bacteria and maintained extended positive growth (Supplementary Material 1). Our analysis here has focused on *Bd* as the sole food source and indicates that micrograzer dynamics (growth and ingestion) lead to control of *Bd* populations. These findings argue that *Bd*-host dynamics should now be examined in a wider food-web context, with mixed an assemblage of microgazers sustained by *Bd* and a wider range of natural food sources.

### *Tetrahymena* Grazing on *Bd*

Globally, *Tetrahymena* is common in shallow waters, living near sediments, where it consumes bacteria and other microbes (Simon et al., 2007; Doerder, 2019). These are the same habitats that *Bd* occupies. *Tetrahymena* is also associated with amphibians where it may be an opportunistic ectoparasite (Thompson, 1958; Holz et al., 2015), but possibly also a consumer of *Bd* zoospores as they emerge from sporangia. Considering its habitat and ability to rapidly reproduce on *Bd* alone, we focused on *T. pyriformis’* ingestion of *Bd* zoospores. In contradiction to Schmeller et al. (2014), attempts to stain *Bd* zoospores with calcofluor-white were not successful; calcofluor stains chitin (Hageage and Harrington, 1984), and although *Bd* sporangia have a chitin wall, zoospores do not (Berger et al., 2005). Therefore, this staining method seems inappropriate for *Bd* zoospores. We then explored vital stains (e.g., cell tracker green), but again we were not successful. Consequently, ingestion estimates relied on the uptake of fluorescent beads as surrogates for *Bd*, which may underestimate rates (e.g., Montagnes and Lessard, 1999). We, therefore, see our predictions as conservative, which we partially account for by examining slightly superior grazers (Figure 6). From our data, a clear Type II functional response was obtained for *T. pyriformis* (Figure 4), providing essential parameters for modeling *Bd*-micrograzer dynamics (see section “Methods”). To our knowledge, this is the first time a functional response on *Bd* sized particles has been obtained for any *Tetrahymena* species: the estimates of *I*_*max*_ and *k* are within the range of those obtained for other ciliates, although the *k*-values are on the lower end of the spectrum (Jürgens and Šimek, 2000; Gismervik, 2005; Pritchard et al., 2016), suggesting *Tetrahymena* has a high affinity for *Bd* size particles; our modeling, therefore, includes micrograzers that are inferior to *Tetrahymena*.

### Modeling Micrograzer Impacts on *Bd* Populations

Empirical evidence suggests that *Paramecium* can reduce *Bd* infections, through examining end point estimates of host infection (Schmeller et al., 2014). Here we explore the temporal dynamics of such control and the potential for micrograzers to prevent host death. Through sensitivity analysis we examine a range of grazers from those with slightly higher grazing rates than *Tetrahymena* – reflecting potential discrimination of beads – to those substantially inferior – reflecting species that may not grow as well on *Bd*, such as *Paramecium*. Our analysis is reductionist and hence more qualitative than quantitative in its predictions. However, it clearly reveals that by applying plausible parameters for both the parasite and micrograzer, in a local environment, chytridiomycosis may at times be prevented and *Bd* virtually eradicated, or at least reduced to negligible levels (Figure 6). Critically, it suggests the time scales over which such dynamics may occur. Admittedly, we indicate that micrograzers that are inferior to *T. pyriformis* are less likely to prevent host death, yet they still, ultimately, reduce *Bd* populations to negligible numbers, potentially preventing further disease outbreaks (Figure 6). Our model, therefore, provides a mechanism to evaluate *Bd*-micrograzer dynamics, and its predictions strongly argue for the continued exploration of micrograzers in *Bd* research, specifically, and in the control of a range of diseases that spread through zoospores or other similarly sized dispersal stages (Kagami et al., 2014; Grossart and Rojas-Jimenez, 2016; Frenken et al., 2019).

To date, models of *Bd*-dynamics (Mitchell et al., 2008; Woodhams et al., 2008; Briggs et al., 2010; Louca et al., 2014) have not included estimates of spore loss by micrograzers. As indicated above, the modeling provided here is instructive and could benefit from elaboration. Given the ubiquity of protozoa in natural waters (Fenchel, 1987) and their potential impact (Figure 6; Kagami et al., 2014; Grossart and Rojas-Jimenez, 2016; Frenken et al., 2019), we suggest there is now a need for better parameterization of micrograzer-*Bd* responses. We suggest three main directions. First, micrograzers, and specifically *Tetrahymena*, exhibit chemosensory behavior (Leick and Hellung-Larsen, 1992); the extent to which protozoa are attracted to amphibian hosts and *Bd* requires evaluation. Second, as indicated above, the role of *Bd* as a supplement rather than a sole dietary component deserves attention. Finally, the Rosenzweig-MacArthur predator-prey model, which we used, has limitations. Model structures such as the independent response model (Fenton et al., 2010) that rely, independently, on growth and ingestion responses provide better predictions (Li and Montagnes, 2015). To this end, we suggest that both functional (ingestion) and numerical (growth) responses associated with *Bd* abundance are established for a range of micrograzers.

### Future Directions for Microbial Ecology and *Bd*

Both *Tetrahymena* and *Paramecium* are common species in shallow waters (Görtz, 1988; Simon et al., 2007; Doerder, 2019) that, as we have shown, are capable of growth on *Bd* zoospores for limited to extended times. Undoubtedly, other protozoa will also consume and grow on *Bd* zoospores. We, therefore, suggest that the role of micrograzers is considered when evaluating *Bd*-dynamics and the dynamics of other zoosporic diseases. For instance, micrograzers may be important in ingesting chytrid spores that infect phytoplankton (Frenken et al., 2020b). However, planktonic levels of chytrid spores associated with phytoplankton blooms are on the order of 10^3^ ml^–1^ (Frenken et al., 2020a), which is at the lowest end of the functional response of *Tetrahymena* (Figure 3), suggesting low ingestion rates for this and related taxa. Instead, other grazers may play greater roles, although the potential control of phytoplankton chytrid epidemics may generally be relatively low, except for the fastest growing, and smallest, grazers such as ciliates and rotifers (Frenken et al., 2020a).

Contributions of micrograzers to disease dynamics are also likely to have a highly site-specific component, due to their dependence on environmental factors (Doerder, 2019). For instance, chytridiomycosis is more prevalent at higher altitudes (Walker et al., 2010), which will often be both cooler and oligotrophic. While temperature may, in part, determine infection burdens (Cohen et al., 2019), there will likely be an interaction with the trophic status of the water. If in oligotrophic waters bacteria are reduced below levels sufficient to support ciliates (<10^6^ ml^–1^), top-down control may be absent, and our analysis suggests that *Bd* may thrive, resulting in chytridiomycosis. Consequently, assessing the abundance of micrograzers in waters where chytridiomycosis occurs or is predicted seems warranted.

Finally, as both *Tetrahymena* and *Paramecium* are already common if not ubiquitous in aquatic environments and are simple and inexpensive to grow in large quantities (Görtz, 1988; Simon et al., 2007), they may be tractable target species for biomanipulation. We support previous suggestions (Schmeller et al., 2014) that by augmenting natural densities of these species, through addition or supplementary feeding, it may be possible to reduce zoospore densities for *Bd in situ.* However, such manipulations could have detrimental, ecosystem-changing effects (Symondson et al., 2002). Extensive evaluation of the role of microgazers in a wider context is, therefore, required before implementation of such approaches.

## Data Availability Statement

The data supporting the conclusions of this article have been made available by the authors, without reservation. They can be found at: https://doi.org/10.6084/m9.figshare.13516577.v1.

## Author Contributions

HF, JJ, and AH conducted the laboratory work, under the guidance of DM. HF, AF, DD, and DM devised and conducted the modeling. TG and MF provided specific guidance on host and chytrid biology, respectively. All authors provided essential input into the conceptualisation of the study, design of the experiments, contributed to writing, and revising the manuscript.

## Conflict of Interest

The authors declare that the research was conducted in the absence of any commercial or financial relationships that could be construed as a potential conflict of interest.
