# Supplementary material for: Microbial Grazers May Aid in Controlling Infections Caused by the Aquatic Zoosporic Fungus Batrachochytrium dendrobatidis
Source: Front Microbiol. 2021 Jan 21;11:592286. doi: 10.3389/fmicb.2020.592286 (PMC7858660; doi:10.3389/fmicb.2020.592286)
Supplement: Supplementary file 1 [file Data_Sheet_1.DOCX]

***Paramecium* growth when fed *Bd* zoospores and bacteria**

***Introduction***

*Paramecium* species seemed to initially survive on zoospores of *Batrachochytrium dendrobatidis* (*Bd*) (Fig. 3, main text). We, therefore, briefly explored the potential for zoospores to supplement a natural bacterial diet. To do so, we compared *Paramecium caudatum* growth with zoospores and bacteria to when no zoospores were present and then compared these data to our previous data on *P. caudatum* growth rate on bacteria alone (Salt et al., 2017). We emphasise that these are additional data and are preliminary. They do, however, suggest that including zoospores in a ciliate’s diet may improve its growth rate. These results encourage further experiments in this direction, given that micrograzers may aid in controlling *Bd* populations (see main text).

***Methods***

Experiments to measure growth rate (*r*, d^-1^) with and without prey followed those in the main text but without the addition of antibiotics, allowing growth of a natural assemblage of bacteria when *Bd* zoospores were present. Experiments were conducted only on *Paramecium caudatum*, for which we have previous data (same strain) growing on bacteria under similar conditions to the ones in this study, except at 20 °C (Salt et al., 2017).

***Results and Discussion***

When feeding on *Bd* zoospores with bacterial prey, *P. caudatum* sustained a positive growth rate of 0.55 ± 0.06 (SE) d^-1^, which was at times higher than when *P. caudatum* was grown on only bacteria (Fig. S1). When no prey were present growth rate was negative.

Previously, it has been shown that *Paramecium* species may consume *Bd* zoospores and reduce their viability in semi-natural mesocosms (Schmeller et al., 2014). Our data (main text) indicate that *Paramecium* cannot survive on zoospores alone for extended periods. However, the results from our supplemental study suggest that in the experiments conducted by Schmeller et al. (2014). *Paramecium* was not just consuming zoospores. *Paramecium* was likely augmenting its diet with natural bacteria and was thus able to grow rapidly and exploit the *Bd* population, likely following dynamics similar to those that we show for *Tetrahymena* (see main text). Further evaluation of a range of natural food sources in combination with *Bd* zoospores is clearly needed to further assess microgazer potential impacts on *Bd* in natural conditions.

**Supplementary Figure 1.** Growth rates (*r*, d^-1^) of three replicates of *Paramecium caudatum* with *Bd* zoospores and bacteria (black lines and symbols) and without any prey (red lines and symbols). The thick, solid black line represents the average growth rate (dashed lines are one SE) of *P. caudatum* in only bacteria obtained from our previous data under virtually identical conditions (Salt et al., 2017).

References

Salt JL, Bulit C, Zhang W, Qi H, Montagnes DJS. Spatial extinction or persistence: landscape‐temperature interactions perturb predator–prey dynamics*.* Ecography. 2017; 40: 1177-86.

Schmeller DS, Blooi M, Martel A, Garner TW, Fisher MC, Azemar F, et al. Microscopic aquatic predators strongly affect infection dynamics of a globally emerged pathogen*.* Curr Biol. 2014; 24: 176-80.
